# Harnessing the MYB-dependent TAL1 5’super-enhancer for targeted therapy in T-ALL

**DOI:** 10.1186/s12943-022-01701-x

**Published:** 2023-01-18

**Authors:** Charlotte Smith, Aurore Touzart, Mathieu Simonin, Christine Tran-Quang, Guillaume Hypolite, Mehdi Latiri, Guillaume P. Andrieu, Estelle Balducci, Marie-Émilie Dourthe, Ashish Goyal, Françoise Huguet, Arnaud Petit, Norbert Ifrah, André Baruchel, Hervé Dombret, Elizabeth Macintyre, Christoph Plass, Jacques Ghysdael, Nicolas Boissel, Vahid Asnafi

**Affiliations:** 1 Université de Paris Cité, Institut Necker Enfants-Malades INEM, Institut National de La Santé Et de La Recherche Médicale (Inserm), U1151 Paris, France; 2 Laboratory of Hematology, Assistance Publique-Hôpitaux de Paris, Hôpital Necker Enfants-Malades 75743, Paris, France; 3 Institut Curie, Orsay, France; 4 CNRS UMR3348, Institut Curie, Orsay, France; 5 INSERM U1278, Centre Universitaire, Orsay, France; 6 PSL Research University, Paris, France; 7 University Paris-Saclay, 91400 Orsay, France; 8 Department of Pediatric Hematology and Immunology, Assistance Publique-Hôpitaux de Paris (AP-HP), Robert Debré Hospital, Université de Paris Cité, Paris, France; 9 Division of Cancer Epigenomics, German Cancer Research Center (DKFZ), 69120 Heidelberg, Germany; 10 Centre Hospitalier Universitaire de Toulouse, Institut Universitaire du Cancer de Toulouse Oncopole, Laboratoire d’Hématologie, Toulouse, France; 11 Service d’Hématologie Et d’Oncologie Pédiatrique, AP-HP, Hôpital Armand Trousseau, Sorbonne Université, Paris, France; 12 UFR Santé, Université Angers, PRES LUNAM, Centre Hospitalier-Universitaire (CHU) d’Angers, Service Des Maladies du Sang Et INSERM U892, 49933 Angers, France; 13 Université de Paris Cité, Institut Universitaire d’Hématologie, EA-3518, Assistance Publique-Hôpitaux de Paris, University Hospital Saint-Louis, Paris, France; 14 German Cancer Research Consortium (DKTK), 69120 Heidelberg, Germany

**Keywords:** Super-enhancer, Oncogene, Targeted therapy, Cancer

## Abstract

The acquisition of genetic abnormalities engendering oncogene dysregulation underpins cancer development. Certain proto-oncogenes possess several dysregulation mechanisms, yet how each mechanism impacts clinical outcome is unclear. Using T-cell acute lymphoblastic leukemia (T-ALL) as an example, we show that patients harboring 5’super-enhancer (5’SE) mutations of the *TAL1* oncogene identifies a specific patient subgroup with poor prognosis irrespective of the level of oncogene dysregulation. Remarkably, the MYB dependent oncogenic 5’SE can be targeted using Mebendazole to induce MYB protein degradation and T-ALL cell death. Of note Mebendazole treatment demonstrated efficacy in vivo in T-ALL preclinical models. Our work provides proof of concept that within a specific oncogene driven cancer, the mechanism of oncogene dysregulation rather than the oncogene itself can identify clinically distinct patient subgroups and pave the way for future super-enhancer targeting therapy.

**Supplementary Information:**

The online version contains supplementary material available at 10.1186/s12943-022-01701-x.

## Background

For many years a major focus of cancer research has been the identification of genetic alterations leading to oncogene dysregulation. Crucially, this has led to the discovery of several targeted therapies such as the tyrosine kinase inhibitor Imatinib in Chronic Myeloid Leukemia (CML), therapies targeting HER2 receptor in breast cancers or BRAF V600E inhibitors in melanoma. Recent studies have evidenced that certain oncogenes have several distinct dysregulation mechanisms, including mutations in non-coding intergenic regions causing ectopic super-enhancer activation [1–3]. Whether different molecular mechanisms affecting oncogene dysregulation might have clinical implications remains unclear. An illustration of this would be the *TAL1* (T-cell Acute Lymphocytic Leukemia Protein 1) gene. *TAL1* is a major transcription factor dysregulated in more than 50% of T-ALL [4]. Although some effort to determine the clinical impact of *TAL1* dysregulation in T-ALL has been made, conclusions remain contradictory [5, 6]. Like several oncogenes, *TAL1* can be overexpressed by chromosomal rearrangement placing its expression under the control of strong *cis*-regulatory elements. Most commonly reported is the *SIL-TAL1* fusion transcript resulting from 90 kb interstitial microdeletions fusing the 5’portion of the gene to the 3’region of its neighboring gene *STIL* (*SCL*-interrupting locus) [7]. A much rarer chromosomal rearrangement involves the translocation of *TAL1* into T-cell Receptor Delta (TCRD) and Beta (TCRB) loci accounting for only ~ 1–2% of T-ALL [1]. Besides chromosomal rearrangement, we and others discovered the second most recurrent dysregulation mechanism involving novel intergenic mutations upstream of the *TAL1* promoter that lead to oncogenic super-enhancer formation [1, 2]. These mutations nucleate the formation of the super-enhancer by creating de novo MYB transcription factor binding sites. MYB in turn recruits transcription co-activators and the transcription factor complex in close proximity to the mutation, thus driving aberrant *TAL1* expression. Here, we report a comprehensive study of the clinical importance and prognostic impact of 5’-*TAL1* super-enhancer mutations in T-ALL. We demonstrate proof of concept that the mechanism of oncogene dysregulation rather than oncogene dysregulation itself can have significant clinical implications and that uncovering the molecular basis for oncogene dysregulation can pave the way to new therapeutic targets beyond direct pharmacological inhibition of oncogenes.

## Results

### 5% of T-ALL have 5’TAL1 super-enhancer (5’SE) mutations

**Fig. 1:**
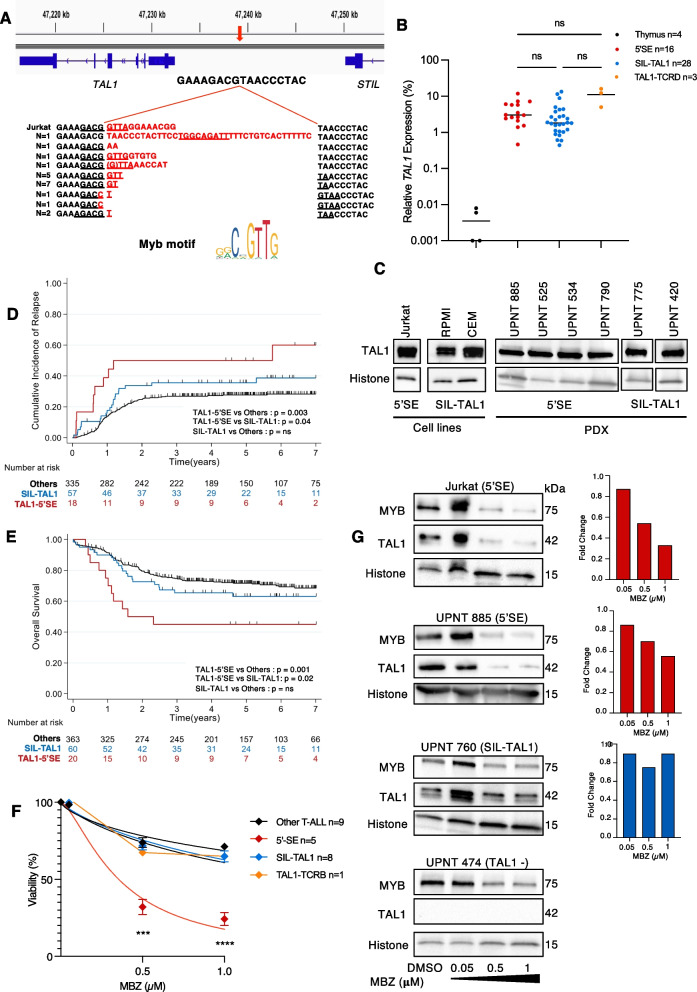
Mebendazole demonstrates anti-leukemic activity in 5’SE T-ALLs with poor clinical outcome due to MYB-mediated TAL1 inhibition. **A** 5’microinsertion sequences aligned to the normal physiological sequence (Hg38). The red arrow denotes the mutation insertion site. All *TAL1* super*-*enhancer mutations introduce de novo MYB binding sites (underlined). **B** The relative *TAL1* expression normalized to *ABL* + *GAPDH* expression in the thymus and T-ALL patients. Kruskal–Wallis; 5’SE *vs.* SIL-TAL1 *p-adj* = *0.5*, 5’SE *vs.* TAL1-TCRD *p-adj* = *0.99,* SIL-TAL1 *vs.* TAL1-TCRD *p-adj* = *0.97.* **C** TAL1 protein expression in 5’SE compared with SIL-TAL1 T-ALL. Left Panel T-ALL cell lines, Right Panel PDX. Histone was used as a loading control. **D** Cumulative Incidence of relapse (CIR) of 5’SE, SIL-TAL1 and Other T-ALL. **E** Kaplan Meier depicting overall (OS) survival of 5’SE, SIL-TAL1 and other T-ALL. **F** Viability curves of 5’SE, SIL-TAL1*,* and Other T-ALL (Cell lines + PDX) at increasing Mebendazole concentrations. Viability was normalized to DMSO controls. The Mean and SEM are shown of duplicate samples. (Two-way ANOVA; 5’SE *vs.* SIL-TAL1 and Other T-ALL *p* < *0.0001*). **G** MYB and TAL1 protein expression after 48 h Mebendazole exposure in the Jurkat cell line, a representative 5’SE, SIL-TAL1 and TAL1 negative (TAL1-) PDX with corresponding *TAL1* mRNA expression for TAL1 + T-ALL (Right Panel). *TAL1* expression was normalized to *GAPDH*

The occurrence of 5’SE mutations was assessed in a large cohort of 443 unselected T-ALLs treated in the GRAALL-2003/2005 (Adult) and FRALLE-2000 (Pediatric) clinical trials. Sanger Sequencing identified 20 5’SE mutated patients accounting for 5% of T-ALL. The microinsertions were of variable size but all mapped to the same genomic position as previously reported [1, 2] (1:47,239,295 hg38) and were predicted to create a neomorphic binding site for MYB transcription factor (Fig. 1A). Of note, we observed a comparable level of *TAL1* expression between 5’SE, TAL1-TCRD and SIL-TAL1 rearranged T-ALLs (Fig. 1B, C), however due to the limited number of TAL1-TCRD cases (*n* = 4) preventing robust statistical analysis, we focused subsequent analyses on 5’SE and SIL-TAL cases. As expected, 5’SE mutations, and SIL-TAL1 were both mutually exclusive with other major T-ALL driver oncogenes such as *TLX1, TLX3, CALM-AF10* (Supplementary Tables 1 and 2) [8, 9]. Furthermore, analysis of 5’SE patients’ oncogenetic landscape revealed a similar mutational profile to SIL-TAL1 patients with a low rate of co-mutations (Fig. S1A-B + S2A + B).

### 5’SE patients have poor clinical outcome

Despite 90% of 5'SE patients achieving complete remission (*vs.* 95% of SIL-TAL1 patients; *p* = *0.6*), having similar clinical and biological characteristics (e.g., age, WBC, immunophenotype), and similar early responses (Prednisone response 42% *vs.* 32% respectively; *p* = *0.6*,) and MRD1 assessments at the end of induction (MRD1 > 10^–4^ in 29% *vs*. 43% respectively, *p* = *0.4*), 5'SE patients were significantly associated with poorer clinical outcomes than SIL-TAL1 patients (Supplementary Table 2). 5’SE patients had significantly increased cumulative incidence of relapse (CIR) (5y-CIR; 50% *vs.* 36%; specific hazard ratio (SHR); 2.3, 95% CI [1.03—5.1]; *p* = *0.04*) and shorter overall survival (OS) (5y-OS: 45% *vs*. 63%; Hazard Ratio (HR); 2.5, 95% CI [1.1—5.4]; *p* = *0.02*) compared with SIL-TAL1 patients (Fig. 1D + E, S3A-B).

Poorer clinical outcomes for 5’SE patients were also true when comparing with Other T-ALL, whereas SIL-TAL1 patients’ outcomes were not significantly different from Other T-ALL. 5'SE patients were associated with an increased cumulative incidence of relapse (CIR) (5y-CIR: 50% *vs.* 28%; specific hazard ratio (SHR), 2.8 95% CI (1.4- 5.4); *p* = *0.003*) and a shorter overall survival (OS) (5y-OS: 45% *vs.* 72%; hazard ratio: 2.9, 95%CI (1.5—5.5); *p* = *0.001*) compared to Other T-ALL (Fig. 1D + E, S3A-B).

Our results emphasize the specificity of this T-ALL subgroup as no other major genetic abnormality was associated with the poorer prognosis observed in 5’SE patients (Fig. S1A-B + Supplementary Table 1). Given their prognostic outlook, 5’SE mutated patients should therefore benefit from innovative clinical management.

### Mebendazole demonstrates anti-leukemic activity in 5’SE T-ALL due to MYB-mediated TAL1 degradation

Mebendazole is a readily available and well tolerated anti-helminth drug that has anti-tumoral activity in a wide range of cancers, including in Acute Myeloid Leukemia via a MYB protein degradation mechanism [10]. Mebendazole specifically and significantly reduced the cell viability of 5’SE T-ALLs in vitro and ex vivo (T-ALL cell lines and Patient Derived Xenografts (PDX)) after 48 h exposure (*p* < *0.0001*). 5’SE T-ALLs had an IC50 of 0.35 μM (95% CI [0.29–0.43] n = 5) compared with SIL-TAL1, TAL1-TCRB, and Other T-ALLs, which had IC50s of 1.81 μM (95% CI [1.37–2.78] *n* = 8), 1.66 μM (95% CI [1.05–4.80] *n* = 1) and 2.99 μM (95% CI [2.00–5.61] *n* = 9) respectively (Fig. 1F + S4A + B). As expected, Mebendazole induced MYB degradation and reduced TAL1 mRNA and protein expression in 5’SE T-ALLs but had minimal effect on TAL1 expression in SIL-TAL1 T-ALLs despite MYB degradation (Fig. 1G). These results confirm 5’SE T-ALLs dependency on the MYB-TAL1 axis for their survival and identified a potential specific targeted therapy for 5’SE patients.

### Mebendazole delays tumor progression *in vivo*

**Fig. 2:**
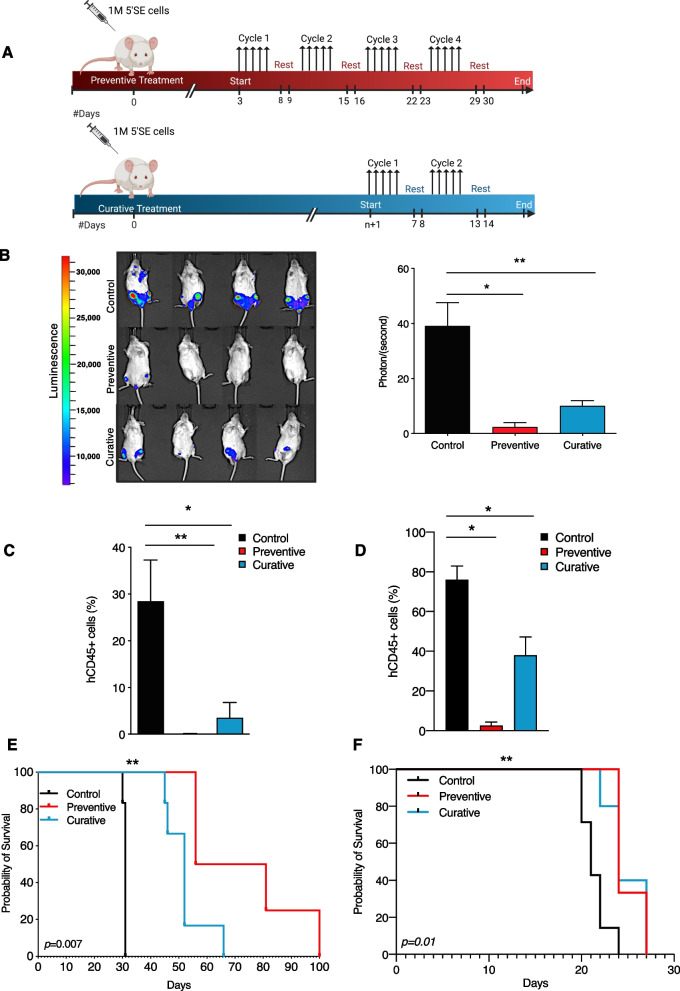
Mebendazole delays tumor progression in 5’SE T-ALLs in vivo. **A** Schematic showing Mebendazole treatment settings. Adapted from “Mouse Experimental Timeline”, by BioRender.com (2022). Retrieved from https://app.biorender.com/biorender-templates. **B** Bioluminescence imaging of NSG recipient mice 31 days after injection with Jurkat cells. Representative images are shown. Mann–Whitney; Control *vs.* Preventive *p* = *0.03,* Control *vs.* Curative *p* = *0.01*. The Mean and SEM are shown*.* **C** hCD45 staining of bone marrow cells 28 days after injection with Jurkat luciferase expressing cells. Mann–Whitney; Control *vs.* Preventive *p* = *0.01,* Control *vs.* Curative *p* = *0.02.* The mean and SEM are shown. **D** Flow Cytometric peripheral blood staining of hCD45 21 days after injection with 5’SE PDX cells. Control *vs*. Preventive *p* = *0.03*, Control *vs.* Curative *p* = *0.02.* The mean and SEM are shown*.* **E** Kaplan Meier survival curves for Control, Preventive and Curative mice. Log-rank (Mantel-Cox) Test; Control *vs.* Preventive *vs.* Curative *p* = *0.007,* Control *vs.* Preventive *p* = *0.005,* Control *vs.* Curative *p* = *0.001*. **F** Kaplan Meier survival curves for Control, Preventive and Curative 5’SE PDX mice. Log-rank (Mantel-Cox) Test; Control *vs.* Preventive *vs.* Curative *p* = *0.004*, Control *vs.* Preventive *p* = *0.009,* Control *vs.* Curative *p* = *0.01*

To test Mebendazole’s ability to hinder leukemic progression in vivo, we injected NSG-mice with Jurkat cells carrying a native 12 bp 5’SE microinsertion [1] that we transduced to express the luciferase gene. Mice were administered Mebendazole in preventive and curative settings (Fig. 2A). Treatment with Mebendazole delayed tumor progression in treated mice compared to control mice. Significantly less bioluminescence, reflecting bulk leukemic engraftment was detected in preventive (*p* = *0.03*) and curative mice (*p* = *0.01*) compared to vehicle control (Fig. 2B). Likewise, hCD45 staining of bone marrow cells revealed significantly reduced leukemic burden in preventive mice (*p* = *0.01*) and curative mice (*p* = *0.02*) compared to control mice (Fig. 2C). Furthermore, Mebendazole improved the overall survival of both preventive *(p* = *0.005)* and curative *(p* = *0.001)* treated mice compared to control mice (Fig. 2E). We also tested Mebendazole’s efficacy in a more clinically relevant model using a 5’SE mutated PDX. Following the same treatment settings (Fig. 2A), hCD45 peripheral blood staining similarly showed reduced leukemic burden in treated mice with significantly fewer leukemic blasts detected in preventive (*p* = *0.03*) and curative mice (*p* = *0.02*) compared to control (Fig. 2D). Importantly, Mebendazole administration resulted in a significantly improved survival in both treatment settings (preventive and curative *p* = *0.01*) (Fig. 2F). Collectively these results demonstrate the efficacy of Mebendazole in reducing leukemic burden and delaying leukemic engraftment by specifically targeting MYB-dependent TAL1^+^ T-ALLs.

## Discussion

The work presented here challenges the paradigm of cancer treatment which has hitherto focused on the identification of genetic alterations underlying expression and maintenance of malignant phenotypes. We have shown that the molecular mechanisms engendering oncogene dysregulation, rather than the level of oncogene overexpression itself, can identify subgroups of poor prognosis.

Clinical Management of T-ALL remains a challenge, especially for relapsed/refractory T-ALL which are associated with extremely poor prognosis. Despite a high response rate after first-line therapy, about 20% of pediatric and 40% of adult T-ALL patients will suffer from relapse [11]. Even though clinical testing of targeted therapy has dramatically increased recently, such treatment options are limited for T-ALL due to the uniqueness of initiating events and oncogenic drivers implicated in T-ALL leukemogenesis. New approaches are needed for the design of personalized medicines in high-risk T-ALL [12]. Several oncogenes are considered ‘undruggable’ primarily because of their critical functions in developmental and physiological contexts, or because of technical constraints designing specific and efficient molecules [11, 13]. Hence, a void exists in the development of effective personalized medicines for such high-risk T-ALL and oncogene-driven malignancies.

Our study has shown proof-of-concept that a mechanism of oncogene dysregulation such as the previously reported 5’SE [1, 2], is associated with poor clinical outcome and can be efficiently targeted to suppress oncogenic signaling. While super-enhancer (SE) dysregulation sustains oncogenesis, it also creates an exploitable vulnerability. Among candidate targets, bromodomain and extra-terminal domain (BET) protein BRD4 has been implicated as a core component of SE activation in cancer. Its targeting has shown promising results in several hematological malignancies [14]. Other critical SE regulators such as CDK7 are currently being evaluated to disrupt SE-driven oncogene dysregulation [15]. In line with this, our study provides a strong rationale for the development of novel therapies targeting the dysregulation mechanism such as SEs to efficiently suppress oncogene driven cancers.

## Conclusions

In this study we have shown that within a specific oncogene driven cancer the underlining molecular mechanism responsible for oncogene dysregulation can have significant clinical implications rather than the level of oncogene overexpression. Importantly, we demonstrate that Mebendazole can be repurposed to induce MYB mediated TAL1 degradation and induce cell death in MYB-dependent 5’SE T-ALLs, highlighting the importance of understanding the molecular basis of oncogene dysregulation which can uncover suitable targets such as the 5’SE, exploitable for the development of targeted therapy.

## Supplementary Information

**Additional file 1.**

**Additional file 2.**

## Data Availability

All data generated or analyzed during this study are included in this published article and its supplementary information files.

## Abbreviations

5’SE: 5’Super-enhancer
CR: Cumulative Incidence of relapse
MRD: Minimal Residual Disease
OS: Overall Survival
PDX: Patient Derived Xenograft
SHR: Specific Hazard Ratio
TAL1: T-cell Acute Lymphocytic Leukemia Protein 1
T-ALL: T-cell Acute Lymphoblastic Leukemia
TCR: T-cell Receptor
WBC: White Blood Count

## References

[1] Navarro J-M, Touzart A, Pradel LC, Loosveld M, Koubi M, Fenouil R (2015). Site- and allele-specific polycomb dysregulation in T-cell leukaemia. Nat Commun.

[2] Mansour MR, Abraham BJ, Anders L, Berezovskaya A, Durbin AD, Etchin J (2014). An Oncogenic Super-Enhancer Formed through Somatic Mutation of a Noncoding Intergenic Element. Science.

[3] Hu S, Qian M, Zhang H, Guo Y, Yang J, Zhao X (2017). Whole-genome noncoding sequence analysis in T-cell acute lymphoblastic leukemia identifies oncogene enhancer mutations. Blood.

[4] Ferrando A a, Herblot S, Palomero T, Hansen M, Hoang T, Fox E a (2004). Biallelic transcriptional activation of oncogenic transcription factors in T-cell acute lymphoblastic leukemia. Oncogene.

[5] Bash RO, Crist WM, Shuster JJ, Link MP, Amylon M, Pullen J (1993). Clinical Features and Outcome of T-Cell Acute Lymphoblastic Leukemia in Childhood With Respect to Alterations at the TAL1 Locus: A Pediatric Oncology Group Study. Blood.

[6] D’Angiò M, Valsecchi MG, Testi AM, Conter V, Nunes V, Parasole R (2015). Clinical features and outcome of SIL/TAL1-positive T-cell acute lymphoblastic leukemia in children and adolescents: a 10-year experience of the AIEOP group. Haematologica.

[7] Brown L, Cheng JT, Chen Q, Siciliano MJ, Crist W, Buchanan G (1990). Site-specific recombination of the tal-1 gene is a common occurrence in human T cell leukemia. EMBO J.

[8] Ferrando AA, Neuberg DS, Staunton J, Loh ML, Huard C, Raimondi SC (2002). Gene expression signatures define novel oncogenic pathways in T cell acute lymphoblastic leukemia. Cancer Cell.

[9] Soulier J, Clappier E, Cayuela JM, Regnault A, García-Peydró M, Dombret H (2005). HOXA genes are included in genetic and biologic networks defining human acute T-cell leukemia (T-ALL). Blood.

[10] Walf-Vorderwulbecke V, Pearce K, Brooks T, Hubank M, van den Heuvel-Eibrink MM, Zwaan CM (2018). Targeting acute myeloid leukemia by drug-induced c-MYB degradation. Leukemia.

[11] Desjonquères A, Chevallier P, Thomas X, Huguet F, Leguay T, Bernard M (2016). Acute lymphoblastic leukemia relapsing after first-line pediatric-inspired therapy: a retrospective GRAALL study. Blood Cancer J.

[12] Cordo V, van der Zwet JCG, Canté-Barrett K, Pieters R, Meijerink JPP (2021). T-cell Acute Lymphoblastic Leukemia: A Roadmap to Targeted Therapies. Blood Cancer Discov.

[13] Bayón-Calderón F, Toribio ML, González-García S (2020). Facts and Challenges in Immunotherapy for T-Cell Acute Lymphoblastic Leukemia. Int J Mol Sci.

[14] Andrieu GP, Kohn M, Simonin M, Smith C, Cieslak A, Dourthe M-E (2021). PRC2 loss of function confers a targetable vulnerability to BET proteins in T-ALL. Blood.

[15] Kwiatkowski N, Zhang T, Rahl PB, Abraham BJ, Reddy J, Ficarro SB (2014). Targeting transcription regulation in cancer with a covalent CDK7 inhibitor. Nature.

