# Supplementary material for: Harnessing the MYB-dependent TAL1 5’super-enhancer for targeted therapy in T-ALL
Source: Mol Cancer. 2023 Jan 18;22:12. doi: 10.1186/s12943-022-01701-x (PMC9847025; doi:10.1186/s12943-022-01701-x)
Supplement: Supplementary file 1 — Additional file 1. [file 12943_2022_1701_MOESM1_ESM.pdf]

# Supplementary Information

| Gene | 5'SE (% of alteration) | SIL-TAL1 (% of alteration) | Other (% of aleration) | 5'SE *vs.* Other (Fisher Test) | 5'SE vs SIL-TAL1 (Fisher Test) |
|---|---|---|---|---|---|
| ***AKT1*** | 0 | 0 | 1 | *1,000* | *1,000* |
| ***ASXL1*** | 6 | 4 | 5 | *0,609* | *0,567* |
| ***BCL11B*** | 17 | 7 | 19 | *1,000* | *0,348* |
| ***CDKN2A*** | 100 | 86 | 66 | ***0,001*** | *0,186* |
| ***CTCF*** | 0 | 2 | 6 | *0,612* | *1,000* |
| ***DNM2*** | 0 | 0 | 19 | *0,052* | *1,000* |
| ***DNMT3A*** | 0 | 0 | 5 | *1,000* | *1,000* |
| ***EED*** | 0 | 2 | 5 | *1,000* | *1,000* |
| ***EP300*** | 0 | 2 | 5 | *1,000* | *1,000* |
| ***ETV6*** | 0 | 0 | 4 | *1,000* | *1,000* |
| ***EZH2*** | 0 | 4 | 9 | *0,384* | *1,000* |
| ***FBXW7*** | 11 | 16 | 21 | *0,386* | *1,000* |
| ***FLT3*** | 0 | 0 | 1 | *1,000* | *1,000* |
| ***GATA3*** | 0 | 0 | 1 | *1,000* | *1,000* |
| ***IDH1*** | 0 | 0 | 1 | *1,000* | *1,000* |
| ***IDH2*** | 6 | 0 | 2 | *0,337* | *0,240* |
| ***IKZF1*** | 0 | 4 | 7 | *0,617* | *1,000* |
| ***IL7R*** | 6 | 0 | 12 | *0,706* | *0,240* |
| ***JAK1*** | 0 | 2 | 8 | *0,381* | *1,000* |
| ***JAK3*** | 6 | 4 | 19 | *0,216* | *0,567* |
| ***KMT2A*** | 0 | 5 | 3 | *1,000* | *1,000* |

| Gene | 5'SE (% of alteration) | SIL-TAL1 (% of alteration) | Other (% of aleration) | 5'SE *vs.* Other (Fisher Test) | 5'SE *vs.* SIL-TAL1 (Fisher Test) |
|---|---|---|---|---|---|
| ***KMT2D*** | 0 | 2 | 4 | *1,000* | *1,000* |
| ***KRAS*** | 0 | 0 | 5 | *1,000* | *1,000* |
| ***LEF1*** | 17 | 19 | 12 | *0,459* | *1,000* |
| ***NF1*** | 6 | 2 | 10 | *1,000* | *0,425* |
| ***NOTCH1*** | 39 | 56 | 76 | ***0,001*** | *0,280* |
| ***NRAS*** | 6 | 0 | 10 | *1,000* | *0,240* |
| ***PHF6*** | 0 | 7 | 39 | ***< 0.001*** | *0,567* |
| ***PIK3CA*** | 6 | 0 | 2 | *0,337* | *0,240* |
| ***PIK3R1*** | 6 | 7 | 3 | *0,433* | *1,000* |
| ***PTEN*** | 17 | 49 | 10 | *0,418* | ***0,026*** |
| ***PTPN11*** | 0 | 2 | 1 | *1,000* | *1,000* |
| ***PTPN2*** | 0 | 2 | 9 | *0,382* | *1,000* |
| ***RUNX1*** | 0 | 0 | 8 | *0,380* | *1,000* |
| ***SETD2*** | 0 | 0 | 5 | *1,000* | *1,000* |
| ***SH2B3*** | 0 | 0 | 5 | *1,000* | *1,000* |
| ***STAT5B*** | 11 | 0 | 8 | *0,659* | *0,055* |
| ***SUZ12*** | 6 | 2 | 16 | *0,328* | *0,425* |
| ***TET2*** | 11 | 4 | 2 | *0,083* | *0,242* |
| ***TET3*** | 0 | 4 | 2 | *1,000* | *1,000* |
| ***TP53*** | **0** | **2** | **5** | ***1,000*** | ***1,000*** |
| ***WT1*** | 0 | 0 | 12 | *0,239* | *1,000* |

**Supplementary Table 1. Frequency of gene alterations according to TAL1 status**

Genes in red have been reported as associated with poor prognosis in T-ALL

| Total: **n = 443**<br>Variable | **n = 60** (14%)<br>SIL-TAL1 | **SIL-TAL1 *vs.* 5'SE**<br>*p-value*[2] | **n = 20** (5%)<br>5'SE | **5'SE *vs.* Other T-ALL**<br>*p-value*[2] | **n = 363**<br>Other T-ALLs |
|---|---|---|---|---|---|
| Male | 47 / 60 (78%) | >0.9 | 16 / 20 (80%) | 0.6 | 265 / 363 (73%) |
| Age (y)[1] | 12.8 (3.0-51.8) | 0.5 | 12.0 (2.8-27.7) | **0.015** | 16.5 (1.1-59.1) |
| WBC (G/l)[1] | 165 (4-770) | 0.15 | 250 (26-980) | **<0.001** | 47 (0-788) |
| CNS Involvement | 7 / 59 (12%) | 0.7 | 3 / 20 (15%) | 0.5 | 40 / 361 (11%) |
| **Immunophenotype** | | | | | |
| ETP phenotype | 1 / 39 (3%) | >0.9 | 0 / 11 (0%) | 0.13 | 52 / 255 (20%) |
| Immature (IM0/δ/γ) | 0 / 53 (0%) | 0.3 | 1 / 18 (6%) | 0.09 | 83 / 325 (26%) |
| Cortical (IMB, preαβ) | 29 / 53 (55%) | 0.4 | 12 / 18 (67%) | 0.15 | 156 / 325 (48%) |
| Mature TCRαβ | 24 / 53 (45%) | 0.3 | 5 / 18 (28%) | 0.07 | 39 / 325 (12%) |
| αβ lineage | 53 / 53 (100%) | 0.2 | 17 / 18 (94%) | **0.002** | 195 / 325 (60%) |
| Mature TCRγδ | 0 / 53 (0%) | >0.9 | 0 / 18 (0%) | 0.15 | 47 / 325 (14%) |
| **Oncogenetic classification** | | | | | |
| TLX1 | 0 / 60 (0%) | >0.9 | 0 / 20 (0%) | 0.09 | 49 / 321 (15%) |
| TLX3 | 0 / 60 (0%) | >0.9 | 0 / 20 (0%) | **0.02** | 68 / 321 (21%) |
| CALM-AF10 | 0 / 60 (0%) | >0.9 | 0 / 20 (0%) | >0.9 | 11 / 321 (3%) |
| High Risk Classifier | 39 / 58 (67%) | 0.6 | 12 / 20 (60%) | 0.10 | 145 / 360 (40%) |
| **Treatment Response** | | | | | |
| Prednisone response | 18 / 56 (32%) | 0.6 | 8 / 19 (42%) | 0.2 | 203 / 359 (57%) |
| Chemosensitivity | 54 / 59 (92%) | 0.11 | 15 / 20 (75%) | 0.6 | 238 / 355 (67%) |
| MRD1 > 10-4 | 21 / 49 (43%) | 0.4 | 5 / 17 (29%) | 0.5 | 103 / 256 (40%) |
| Complete Remission | 57 / 60 (95%) | 0.6 | 18 / 20 (90%) | 0.7 | 335 / 363 (92%) |
| Allograft | 7 / 59 (12%) | 0.7 | 1 / 19 (5%) | **0.03** | 97 / 347 (28%) |
| **Outcome** | | | | | |
| 5-year CIR [95% CI] | 36% [25;50] | **0.04** | 50% [30;74] | **0.003** | 28% [23;33] |
| 5-year OS [95% CI] | 63% [49;74] | **0.02** | 45% [23;65] | **0.001** | 72% [67;76] |

**Supplementary Table 2. Clinical features of 5'SE mutated T-ALL *vs.* SIL-TAL1 *vs.* Other T-ALL**

[1] Statistics presented: Median (Minimum-Maximum)

[2]Statistical tests performed: Fisher's exact test; Wilcoxon rank-sum test. *p*-values <0.05 are indicated in bold WBC, White blood count; CNS, central nervous system; ETP, early thymic precursor; High Risk classifier, NOTCH1/FBXW7-RAS/ PTEN classifier [1]; CR, complete remission; MRD, minimal residual disease; Allo-HSCT, allogeneic hematopoietic stem cell transplantation; CIR, cumulative incidence of relapse; OS, overall survival, CI: confidence interval.

A

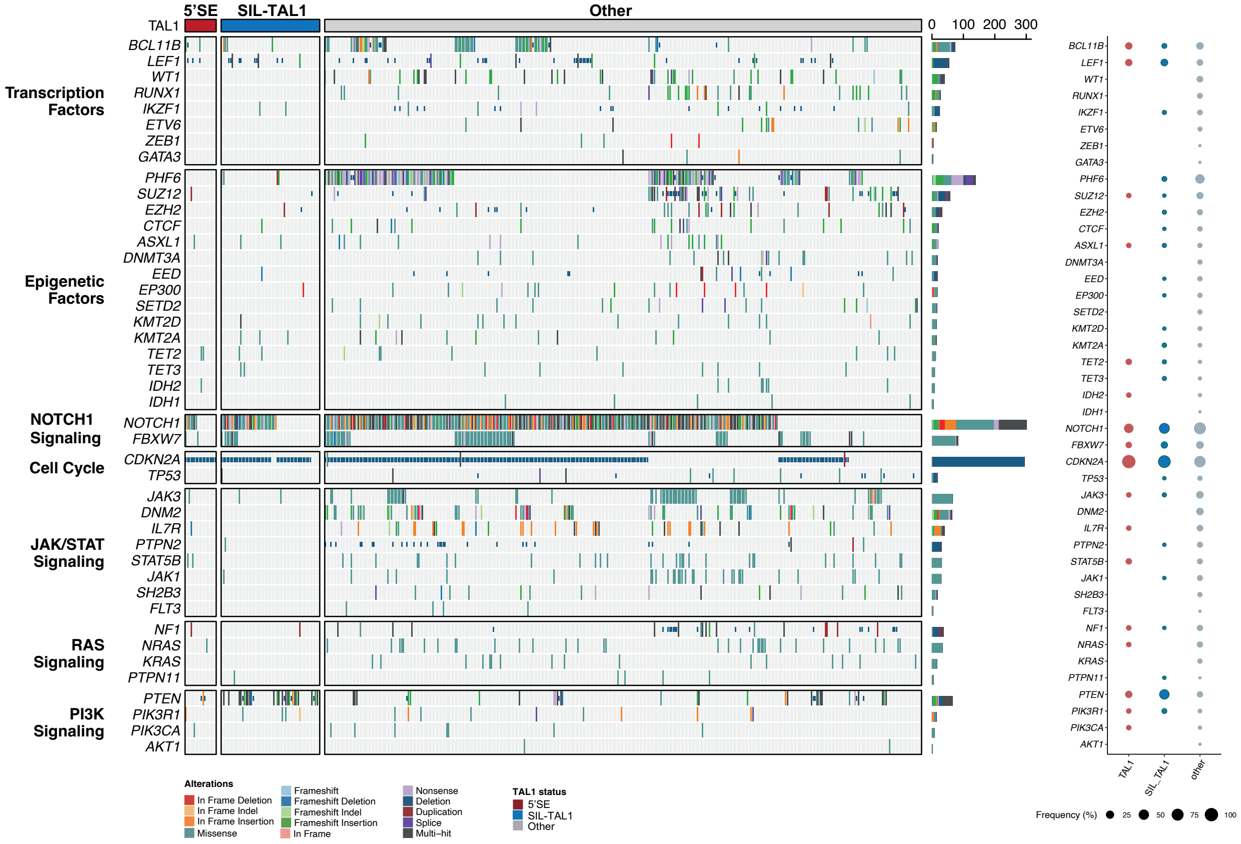
5'SE
SIL-TAL1
Other
TAL1
0 100 200 300
Transcription Factors
BCL11B
LEF1
WT1
RUNX1
IKZF1
ETV6
ZEB1
GATA3
Epigenetic Factors
PHF6
SUZ12
EZH2
CTCF
ASXL1
DNMT3A
EED
EP300
SETD2
KMT2D
KMT2A
TET2
TET3
IDH2
IDH1
NOTCH1 Signaling
NOTCH1
FBXW7
Cell Cycle
CDKN2A
TP53
JAK/STAT Signaling
JAK3
DNM2
IL7R
PTPN2
STAT5B
JAK1
SH2B3
FLT3
RAS Signaling
NF1
NRAS
KRAS
PTPN11
PI3K Signaling
PTEN
PIK3R1
PIK3CA
AKT1
TAL1
SIL_TAL1
other
Alterations
In Frame Deletion
In Frame Indel
In Frame Insertion
Missense
Frameshift
Frameshift Deletion
Frameshift Indel
Frameshift Insertion
In Frame
Nonsense
Deletion
Duplication
Splice
Multi-hit
TAL1 status
5'SE
SIL-TAL1
Other
Frequency (%) 25 50 75 100

B

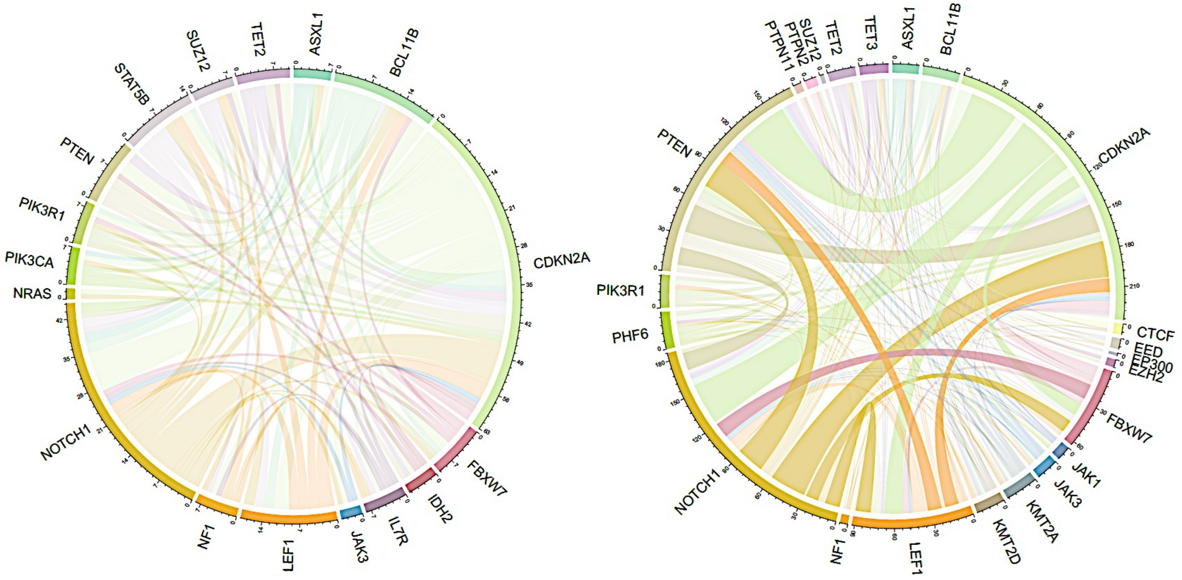
ASXL1
BCL11B
TET2
SUZ12
STAT5B
PTEN
PIK3R1
PIK3CA
NRAS
NOTCH1
NF1
LEF1
JAK3
IL7R
IDH2
FBXW7
CDKN2A
PTPN11
PTPN2
SUZ12
TET2
TET3
ASXL1
BCL11B
CDKN2A
CTCF
EED
EP300
EZH2
FBXW7
JAK1
JAK3
KMT2A
KMT2D
LEF1
NF1
NOTCH1
PHF6
PIK3R1
PTEN

**Fig. S1.**

A) Oncoplot showing the mutation profiles of 5'SE, SIL-TAL1 and other T-ALL and their relative mutation frequencies.

B) Circos plot demonstrating the mutational co-occurrences in 5'SE T-ALL (left) and SIL-TAL1 T-ALL (right).

A

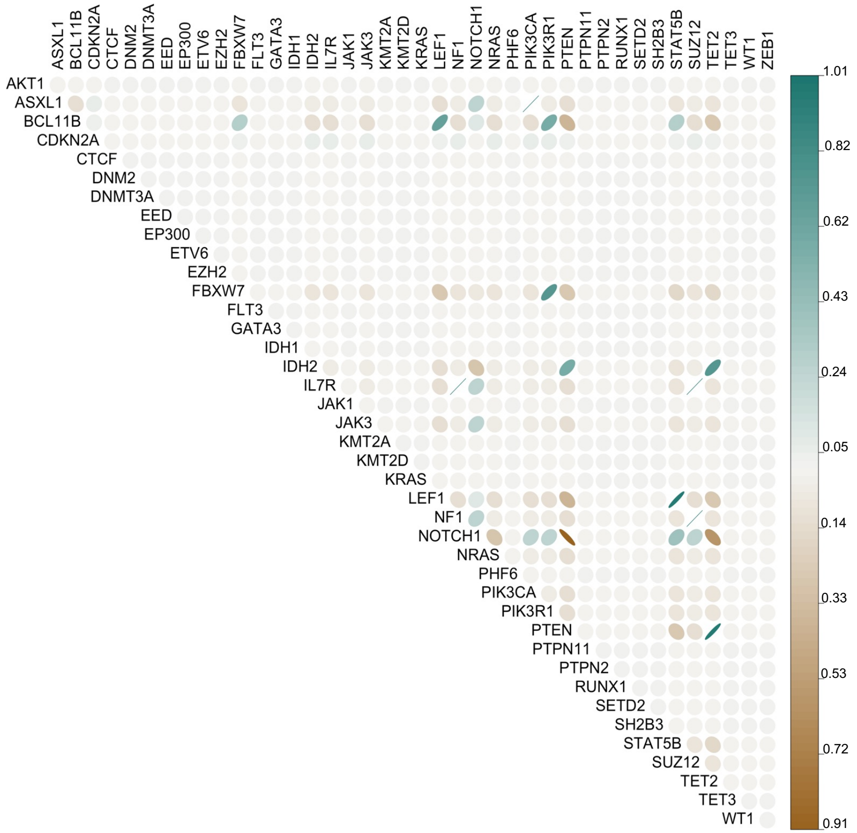
AKT1
ASXL1
BCL11B
CDKN2A
CTCF
DNM2
DNMT3A
EED
EP300
ETV6
EZH2
FBXW7
FLT3
GATA3
IDH1
IDH2
IL7R
JAK1
JAK3
KMT2A
KMT2D
KRAS
LEF1
NF1
NOTCH1
NRAS
PHF6
PIK3CA
PIK3R1
PTEN
PTPN11
PTPN2
RUNX1
SETD2
SH2B3
STAT5B
SUZ12
TET2
TET3
WT1
ZEB1
1.01
0.82
0.62
0.43
0.24
0.05
0.14
0.33
0.53
0.72
0.91

B

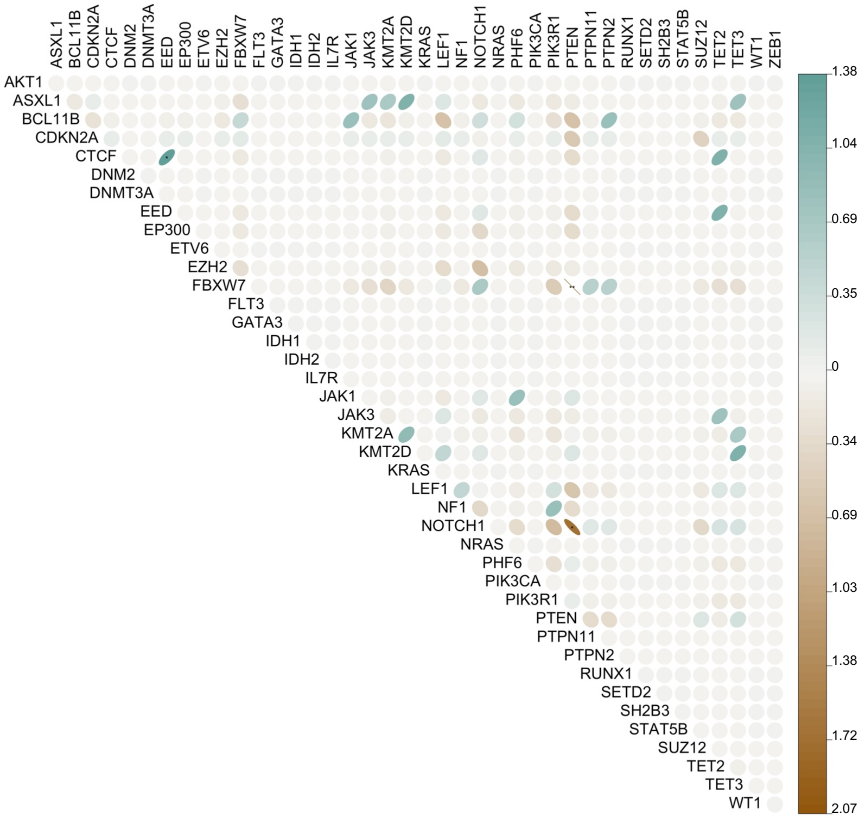
AKT1
ASXL1
BCL11B
CDKN2A
CTCF
DNM2
DNMT3A
EED
EP300
ETV6
EZH2
FBXW7
FLT3
GATA3
IDH1
IDH2
IL7R
JAK1
JAK3
KMT2A
KMT2D
KRAS
LEF1
NF1
NOTCH1
NRAS
PHF6
PIK3CA
PIK3R1
PTEN
PTPN11
PTPN2
RUNX1
SETD2
SH2B3
STAT5B
SUZ12
TET2
TET3
WT1
ZEB1
1.38
1.04
0.69
0.35
0
0.34
0.69
1.03
1.38
1.72
2.07

**Fig. S2**

A) Corroplot showing co-mutations in 5'SE patients and (B) SIL-TAL1 patients. Co-occurrences and mutual exclusions in 5'SE and SIL-TAL1 T-ALL were computed with the DISCOVER algorithm. Strong correlations are indicated by large ellipses whereas weak correlations are indicated by small ellipses. The colors of the scale bar denote the nature of the correlation, with +1 indicating a perfect positive correlation (blue) and -1 indicating a perfect negative correlation (brown) between two genetic alterations.

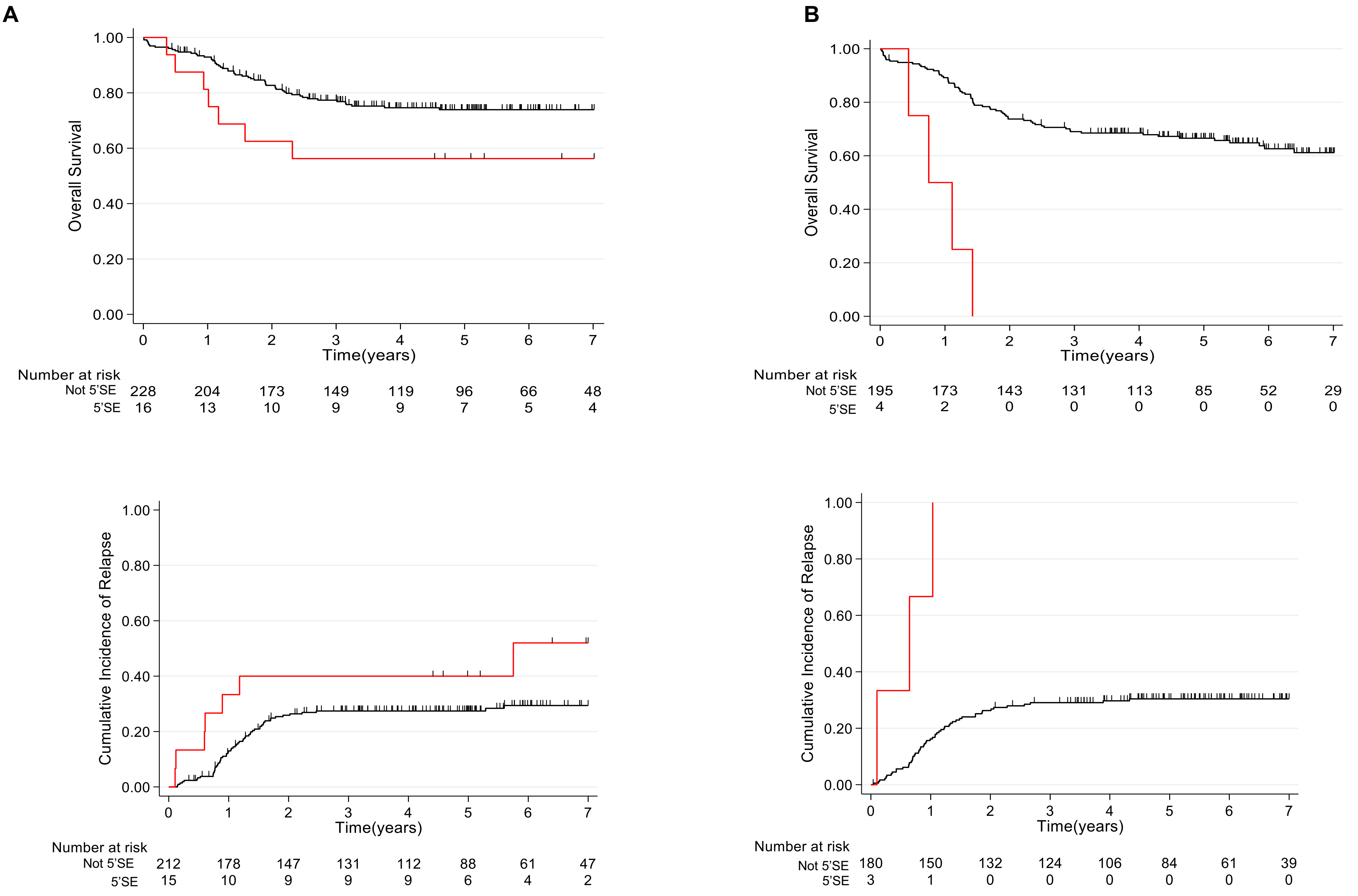
A
B
Overall Survival
Cumulative Incidence of Relapse
Time(years)
Number at risk
Not 5'SE 228 204 173 149 119 96 66 48
5'SE 16 13 10 9 9 7 5 4
Not 5'SE 195 173 143 131 113 85 52 29
5'SE 4 2 0 0 0 0 0 0
Not 5'SE 212 178 147 131 112 88 61 47
5'SE 15 10 9 9 9 6 4 2
Not 5'SE 180 150 132 124 106 84 61 39
5'SE 3 1 0 0 0 0 0 0

**Fig. S3.**

A) Kaplan Meier survival curve (top panel) and cumulative incidence of relapse (bottom panel) for pediatric FRALLE 2000 T-ALL

B) Kaplan Meier survival curve (top panel) and cumulative incidence of relapse (bottom panel) for adult GRAALL 2003-2005 T-ALL

A B

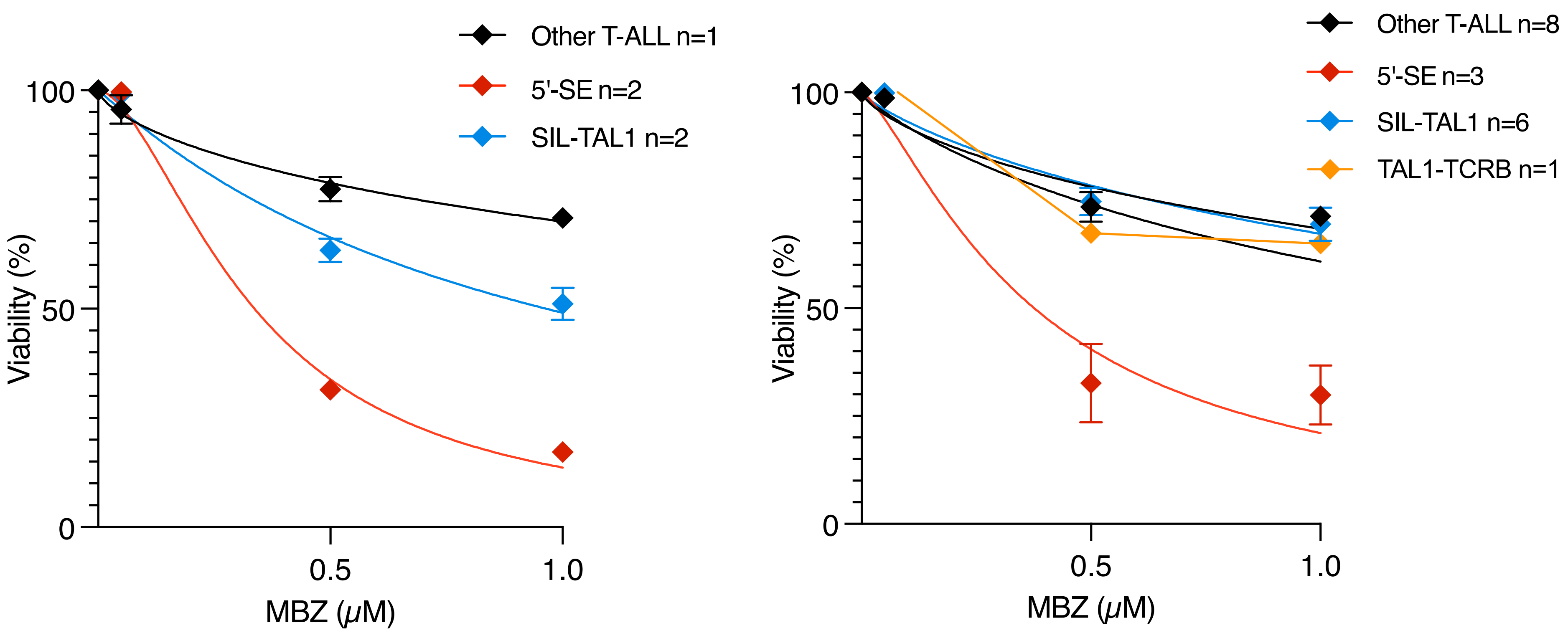

Other T-ALL n=1
5'-SE n=2
SIL-TAL1 n=2
100
50
0
Viability (%)
0.5
1.0
MBZ (μM)
Other T-ALL n=8
5'-SE n=3
SIL-TAL1 n=6
TAL1-TCRB n=1
100
50
0
Viability (%)
0.5
1.0
MBZ (μM)

**Fig S4.**

Viability curves of 5'SE, SIL-TAL1, and Other T-ALL Cell lines (A) and PDX (B) at increasing Mebendazole concentrations. Viability was normalized to DMSO controls. The Mean and SEM are shown of duplicate samples. (Two-way ANOVA; 5'SE *vs.* SIL-TAL1 and Other T-ALL $p<0.0001$. IC50s 5'SE cell lines = 0.34$\mu$M, SIL-TAL1 cell lines – 0.97$\mu$M, Other T-ALL cell line = 3.52$\mu$M. IC50s for 5'SE PDXs = 0.38$\mu$M, SIL-TAL1 PDXs=2.39$\mu$M, TAL1-TCRB PDX = 1.66$\mu M$, Other T-ALL PDXs = 2.94$\mu$M.

## Supplementary References

1 Trinquand A, Tanguy-Schmidt A, Ben Abdelali R, Lambert J, Beldjord K, Lengliné E *et al.* Toward a NOTCH1/FBXW7/RAS/PTEN–Based Oncogenetic Risk Classification of Adult T-Cell Acute Lymphoblastic Leukemia: A Group for Research in Adult Acute Lymphoblastic Leukemia Study. *J Clin Oncol* 2013; **31**: 4333–4342.
