# Supplementary material for: Harnessing the MYB-dependent TAL1 5’super-enhancer for targeted therapy in T-ALL
Source: Mol Cancer. 2023 Jan 18;22:12. doi: 10.1186/s12943-022-01701-x (PMC9847025; doi:10.1186/s12943-022-01701-x)
Supplement: Supplementary file 2 — Additional file 2. [file 12943_2022_1701_MOESM2_ESM.pdf]

Supplementary Materials

Patient samples

443 unselected patient samples from the GRAALL-2003,-2005 trials (#NCT00222027; #NCT00327678) and the FRALLE 2000 trials (199 adult; 244 pediatric) were included in this study based on the availability of tumoral DNA for 5'SE mutation screening (*See Supplementary Table 4*) Blood or bone marrow samples were enriched in more than 80% of leukemic blasts and collected at diagnosis with patients' informed consent. Trials were conducted according to the declaration of Helsinki. The Immunophenotype and oncogenic characteristics were evaluated as previously described [1–5].

Patient Derived xenografts (PDX)

1x10^6 viable primary cells were injected retro-orbitally in 8-week-old immunodeficient NOD/SCIDγc-/- mice. Leukemic burden was evaluated by flow cytometric hCD7+, hCD45+ staining in the peripheral blood. Mice were euthanized when >80% of human leukemic blasts had disseminated the blood or if they demonstrated clinical signs of the disease (Weight loss >10%, neurological symptoms, tumor growth). Primary cells were harvested from the bone marrow (tibiae, femur and vertebrae) for *ex vivo* culture. Animal experimentation was evaluated and approved by the Institute's ethics committee and the Ministère de l'enseignement supérieur de la recherche et de l'innovation. (PROJET APAFIS # 8853 N° 2017020814103710)

In vivo transplantation

In vivo transplantation of jurkat luciferase expressing cells and treatment with Mebendazole took place at the Institut Curie animal facility with 6-7-week-old NOD/SCIDγc-/- mice. Treatment with Mebendazole commenced after a 1-week acclimation period. *In vivo* transplantation of 5'SE PDX cells and treatment with Mebendazole took place at the Institut

Necker Enfants-Malades animal facility with NOD/SCIDγc-/- mice. All experiments were performed in accordance with the animal welfare committee. $1x10^6$ luciferase expressing Jurkat or 5'SE PDX cells were transplanted into NOD/SCIDγc-/- mice by lateral tail injection or retro-orbitally. Treated mice were administered 50 mg/kg of Mebendazole by oral gavage in a suspension of 50:50 PBS/Corn Oil for 5 days followed by 2 days rest (1 cycle). Control mice followed the same 5-2-day treatment cycle but with vehicle (Corn oil) n=6. The preventive treatment group started treatment at day 3 post injection and were treated for 4 cycles (n=4), and the curative treatment group started treatment after the dissemination of leukemic blasts (bioluminescence signal for jurkat-luciferase mice or >1% leukemic blasts detected in the peripheral blood for 5'SE PDX mice) post injection and were treated for 2 cycles (n=6). Leukemic burden was assessed by measuring luciferase activity with the IVIS Spectrum (Perkin-Elmer) and by hCD45 flow cytometric staining of blood and bone marrow samples.

Cell culture and reagents

T-ALL cell lines ATCC® were cultured in RPMI-1640 (Merck R8758) supplemented with 10% fetal bovine serum, 50µg/mL streptomycin, 50 UI penicillin and 4mM L-glutamine. PDX cells were cultured *ex vivo* in RPMI-1640 (Merck R8758) supplemented with 20% fetal bovine serum, 50µg/mL streptomycin, 50 UI penicillin, 4mM L-glutamine and cytokines 50ng/mL hSCF, 20ng/mL hFLT3-L, 10ng/mL hIL7 and 20nM insulin. All cells were cultured at 37°C and in the presence of 5% CO2. Mebendazole (Sigma-Aldrich) was added to cell cultures at increasing concentrations. Cell viability was determined by flow cytometry using Annexin V-APC/ Propidium Iodide (Biolegend) staining 48h after exposure to Mebendazole or DMSO controls.

Western Blot

T-ALL cell lines or PDX cells were washed in cold Phosphate Buffered Saline (PBS) and lysed in RIPA buffer (Cell Signaling Technology) with 50X EDTA-free complete protease inhibitor (Sigma-Aldrich) and PMSF-protease inhibitor (Thermo Scientific). Cell lysates were denatured, separated by SDS-PAGE and transferred onto 0.2µM nitrocellulose membranes (Bio-Rad). Membranes were blocked for 1 hour in either 5%-milk T-PBS or 5%-BSA T-PBS buffer. Membranes were incubated at 4°C overnight in primary antibody cMYB (Abcam Recombinant Anti-c-Myb antibody [EPR718(2)], TAL1 (Merck-Millipore Anti-TAL1 clone BTL73 04-123), or Histone (Cell Signaling Technology Histone H3 (D1H2) rabbit mAB). Membranes were subsequently washed and incubated with the appropriate secondary antibody coupled to HRP for 1 hour. Proteins were detected using chemiluminescence West Dura Super signal™ or Super signal™ West Femto substrates (Thermo Scientific) and visualized using ChemiDoc XRS (Bio-Rad).

RQ-PCR

Total RNA was isolated using RNAeasy Mini kits and treated with DNase I (Invitrogen, Life Technologies). RNA was reverse transcribed with Superscript III (Invitrogen, Life technologies). *TAL1* transcripts were quantified using specific primers (Table S2), Taqman® probes and the ABI Prism 7500 Sequence Detection System (Applied Biosystems). Gene expression was normalized to the *ABL* and h*GAPDH* (Applied Biosystems) housekeeping genes using the ΔCT method (relative expression is equal to $2^{-\Delta\Delta CT}$). Relative fold change in gene expression was calculated using DMSO as control and normalized to *hGAPDH.*

5’SE Screening

5’SE mutations were screened by PCR amplification and Sanger sequencing using specific primers encompassing the microinsertion site as previously described[6] . The primer sequences can be found in *Supplementary Table 4: Primer sequences.*

Gene mutation screening

A custom capture Nextera XT gene panel (Illumina, San Diego, CA) targeting all coding exons and their adjacent splice junctions of 80 genes was designed, based on their presence in hematological neoplasms. The list of genes included in the panel can be found below in *Supplementary Table 3.* DNA Libraries were prepared using Nextera Rapid Capture Enrichment protocol and underwent 2x150bp paired-end sequencing on Illumina MiSeq sequencing system with MiSeq Reagent Kit v2 (Illumina). Briefly, sequence reads were filtered and mapped to the human genome (GRCh37/hg19) using in-house software (Polyweb, Institut Imagine, Paris). Annotated variants were selected after filtering out calls according to the following criteria: (i) coverage < 30x, <10 alternative reads or variant allelic fraction (VAF) <7%; (ii) Polymorphisms described in dbSNP, 1000Genomes, EVS, Gnomad and EXAC with a calculated mean population frequency >0.1%. Non-filtered variants were annotated using somatic database COSMIC (version 78) and ProteinPaint (St Jude Children’s Research Hospital – Pediatric Cancer data portal).

Statistical Analysis

Statistical analyses were either performed with Student *t*-test or ANOVA using GraphPad Prism 9 software. Normality tests were applied to determine if the datasets were suitable for parametric or non-parametric tests. The following symbols were used to indicate significant differences: ns, $p > 0.05$; *, $p \leq 0.05$; **, $p \leq 0.01$; ***, $p \leq 0.001$, $p \leq 0.0001$ ****. All p-values were two-sided.

Comparisons for categorical and continuous variables between subgroups (5'SE, SIL-TAL1, and Other T-ALL) were performed with Fisher's exact test and Mann–Whitney test, respectively. Overall survival (OS) was calculated from the date of diagnosis to the last follow-up date censoring patients alive. The cumulative incidence of relapse (CIR) was calculated from the complete remission date, to the date of relapse censoring patients alive without relapse at the last follow-up date. Relapse and death in complete remission were considered competitive events. Univariate analysis assessing the impact of TAL1 status was performed with a Cox model. Statistical analyses were performed with STATA software (16.1, StataCorp LLC, College Station, TX). All p-values were two-sided, with $p < 0.05$ denoting statistical significance. Oncoplot and Circos plots were generated using R software.

Minimal Residual Disease (MRD)

T-cell receptor TCR gene rearrangement-based evaluation was centrally assessed for patients who reached complete remission after the first induction cycle on bone marrow samples after induction (MRD1). MRD was centrally assessed by real-time quantitative allele-specific oligo-nucleotide PCR and interpreted according to EuroMRD group guidelines [7]

Supplementary Table 3. Next Generation Sequencing targeted panel

Next Generation Sequencing panel (whole exon analysis)							
Gene	ENST	***Gene***	ENST	***Gene***	ENST	***Gene***	ENST
AKT1	ENST00000554581_14	***ECT2L***	ENST00000423192_6	***JAK3***	ENST00000458235_19	***RPL5***	ENST00000370321_1
ASXL1	ENST00000375687_20	***EED***	ENST00000263360_11	***KIT***	ENST00000288135_4	***RUNX1***	ENST00000344691_21
ATM	ENST00000278616_11	***EP300***	ENST00000263253_22	***KMT2A***	ENST00000534358_11	***SAMHD1***	ENST00000262878_20
ATXN1	ENST00000244769_6	***ETV6***	ENST00000396373_12	***KMT2D***	ENST00000301067_12	***SETD2***	ENST00000409792_3
BCL11B	ENST00000345514_14	***EZH2***	ENST00000320356_7	***KRAS***	ENST00000311936_12	***SF3B1***	ENST00000335508_2
BRAF	ENST00000496384_7	***FAS***	ENST00000355740_10	***LEF1***	ENST00000265165_4	***SH2B3***	ENST00000341259_12
CARD11	ENST00000396946_7	***FBXW7***	ENST00000281708_4	***NF1***	ENST00000358273_17	***SKP2***	ENST00000353411_5
CCR4	ENST00000330953_3	***FLT3***	ENST00000241453_13	***NOTCH1***	ENST00000277541_9	***SRSF2***	ENST00000392485_17
CD58	ENST00000457047_1	***FYN***	ENST00000354650_6	***NRAS***	ENST00000369535_1	***STAT3***	ENST00000264657_17
CDKN2A	ENST00000579755_9	***GATA3***	ENST00000379328_10	***PHF6***	ENST00000332070_X	***STAT5B***	ENST00000293328_17
CEBPA	ENST00000498907_19	***HACE1***	ENST00000262903_6	***PIK3CA***	ENST00000263967_3	***STIL***	ENST00000337817_1
CNOT3	ENST00000406403_19	***HIST1H1B***	ENST00000331442_6	***PIK3R1***	ENST00000521381_5	***SUZ12***	ENST00000322652_17
CTCF	ENST00000264010_16	***HNRNPA1***	ENST00000546500_12	***POT1***	ENST00000357628_7	***TAL1***	ENST00000294339_1
CUL3	ENST00000264414_2	***HNRNPR***	ENST00000478691_1	***PTEN***	ENST00000371953_10	***TET2***	ENST00000540549_4
CUX1	ENST00000360264_7	***IDH1***	ENST00000415913_2	***PTPN11***	ENST00000351677_12	***TET3***	ENST00000409262_2
CXCR4	ENST00000409817_2	***IDH2***	ENST00000330062_15	***PTPRC***	ENST00000442510_1	***TP53***	ENST00000420246_17
CXXC4	ENST00000394767_4	***IKZF1***	ENST00000349824_7	***RB1***	ENST00000267163_13	***U2AF1***	ENST00000459639_21
CXXC5	ENST00000302517_5	***IL7R***	ENST00000303115_5	***RELN***	ENST00000428762_7	***WT1***	ENST00000332351_11
DNM2	ENST00000359692_19	***IRF4***	ENST00000380956_6	***RHOA***	ENST00000418115_3	***ZEB1***	ENST00000446923_10
DNMT3A	ENST00000264709_2	***JAK1***	ENST00000342505_1	***RPL10***	ENST00000424325_X	***ZRSR2***	ENST00000307771_X

Supplementary Table 4. Primer Sequences

Primer	Sequence 5'-3'	Application
TAL1-F	ACA-ATC-GAG-TGA-AGA-GGA-GAC-CTT-C	RQ-PCR
TAL1-R	ACG-CCG-CAC-AAC-TTT-GGT-G	RQ-PCR
Taqman® TAL1 probe	Fam-CTA-TGA-GAT-GGA-GAT-TAC-TGA-TG-Tamara	RQ-PCR
ABL-F	TGG-AGA-TAA-CAC-TCT-AAG-CAT-AAC-TAA-AGG-T	RQ-PCR
ABL-R	GAT-GTA-GTT-GCT-TGG-GAC-CCA	RQ-PCR
Taqman® ABL probe	Fam-CCA-TTT-TTG-GTT-TGG-GCT-TCA-CAC-CAT-T-Tamara	RQ-PCR
TAL1 5'SE-F	TAC-CTG-TGC-ACA-GCT-GGA-G	Sanger sequencing
TAL1 5'SE-R	GCT-CTC-CTG-ATT-AGC-ATA-CC	Sanger sequencing

Supplementary References

1 Tesio M, Trinquand A, Ballerini P, Hypolite G, Lhermitte L, Petit A *et al.* Age-related clinical and biological features of PTEN abnormalities in T-cell acute lymphoblastic leukaemia. *Leukemia* 2017; **31**: 2594–2600.

2 Asnafi V, Beldjord K, Boulanger E, Comba B, Le Tutour P, Estienne MH *et al.* Analysis of TCR, pTα, and RAG-1 in T-acute lymphoblastic leukemias improves understanding of early human T-lymphoid lineage commitment. *Blood* 2003; **101**: 2693–2703.

3 Asnafi V, Buzyn A, Le Noir S, Baleydier F, Simon A, Beldjord K *et al.* NOTCH1/FBXW7 mutation identifies a large subgroup with favorable outcome in adult T-cell acute lymphoblastic leukemia (T-ALL): A Group for Research on Adult Acute Lymphoblastic Leukemia (GRAALL) study. *Blood* 2009; **113**: 3918–3924.

4 Bond J, Marchand T, Touzart A, Cieslak A, Trinquand A, Sutton L *et al.* An early

thymic precursor phenotype predicts outcome exclusively in HOXA-overexpressing adult T-cell acute lymphoblastic leukemia: a Group for Research in Adult Acute Lymphoblastic Leukemia study. *Haematologica* 2016; **101**: 732–740.

5 Bergeron J, Clappier E, Radford I, Buzyn A, Millien C, Soler G *et al.* Prognostic and oncogenic relevance of TLX1/HOX11 expression level in T-ALLs. *Blood* 2007; **110**: 2324–2330.

6 Navarro J-M, Touzart A, Pradel LC, Loosveld M, Koubi M, Fenouil R *et al.* Site- and allele-specific polycomb dysregulation in T-cell leukaemia. *Nat Commun* 2015; **6**: 6094.

7 van der Velden VHJ, Cazzaniga G, Schrauder A, Hancock J, Bader P, Panzer-Grumayer ER *et al.* Analysis of minimal residual disease by Ig/TCR gene rearrangements: guidelines for interpretation of real-time quantitative PCR data. *Leukemia* 2007; **21**: 604–611.
